# Health Economic Consequences Associated With COVID-19–Related Delay in Melanoma Diagnosis in Europe

**DOI:** 10.1001/jamanetworkopen.2023.56479

**Published:** 2024-02-16

**Authors:** Lara V. Maul, Dagmar Jamiolkowski, Rebecca A. Lapides, Alina M. Mueller, Axel Hauschild, Claus Garbe, Paul Lorigan, Jeffrey E. Gershenwald, Paolo Antonio Ascierto, Georgina V. Long, Michael Wang-Evers, Richard A. Scolyer, Babak Saravi, Matthias Augustin, Alexander A. Navarini, Stefan Legge, István B. Németh, Ágnes J. Jánosi, Simone Mocellin, Anita Feller, Dieter Manstein, Alexander Zink, Julia-Tatjana Maul, Alessandra Buja, Kaustubh Adhikari, Elisabeth Roider

**Affiliations:** 1 Department of Dermatology, University Hospital of Basel, Basel, Switzerland; 2 Department of Dermatology, University Hospital of Zurich, Zurich, Switzerland; 3 Faculty of Medicine, University of Zurich, Zurich, Switzerland; 4 Division of Pediatric Dermatology, Children’s Hospital Auf der Bult, Hannover, Germany; 5 Robert Larner, MD, College of Medicine at the University of Vermont, Burlington, Vermont; 6 Cutaneous Biology Research Center, Department of Dermatology, Massachusetts General Hospital, Harvard Medical School, Charlestown; 7 Department of Dermatology, University Hospital of Schleswig-Holstein, Campus Kiel, Kiel, Germany; 8 Center for Dermatooncology, Department of Dermatology, Eberhard Karls University of Tuebingen, Tuebingen, Germany; 9 Division of Cancer Sciences, University of Manchester, Manchester, United Kingdom; 10 The Christie NHS Foundation Trust, Manchester, United Kingdom; 11 Department of Surgical Oncology, The University of Texas MD Anderson Cancer Center, Houston; 12 Istituto Nazionale Tumori, IRCCS, Fondazione G Pascale, Naples, Italy; 13 Melanoma Institute Australia, The University of Sydney, Sydney, Australia; 14 Royal North Shore Hospital, Sydney, Australia; 15 Royal Prince Alfred Hospital and NSW Health Pathology, Sydney, Australia; 16 Faculty of Medicine and Health, The University of Sydney, Sydney, Australia; 17 Charles Perkins Centre, The University of Sydney, Sydney, Australia; 18 Department of Orthopedics and Trauma Surgery, Medical Center–University of Freiburg, Germany; 19 Department of Anesthesiology, Perioperative and Pain Medicine, Brigham and Women’s Hospital, Harvard Medical School, Boston, Massachusetts; 20 University Medical Center Hamburg-Eppendorf, Hamburg, Germany; 21 Institute of Law and Economics, University of St Gallen, St Gallen, Switzerland; 22 Department of Dermatology and Allergology, University of Szeged, Szent-Györgyi Albert Medical School, Szeged, Hungary; 23 Soft-Tissue, Peritoneum and Melanoma Surgical Oncology Unit, Istituto Oncologico Veneto – IRCCS, Padua, Italy; 24 Department of Surgery, Oncology and Gastroenterology, University of Padua, Padua, Italy; 25 National Agency for Cancer Registration, University of Zurich, Zurich, Switzerland; 26 Foundation National Institute for Cancer Epidemiology and Registration, University of Zurich, Zurich, Switzerland; 27 Department of Dermatology and Allergy, TUM School of Medicine and Health, Technical University of Munich, Munich, Germany; 28 Department of Cardiac, Thoracic, Vascular Sciences and Public Health, University of Padova, Via Loredan, Padova, Italy; 29 School of Mathematics and Statistics, Faculty of Science, Technology, Engineering and Mathematics, The Open University, Milton Keynes, United Kingdom; 30 Department of Genetics, Evolution and Environment, and UCL Genetics Institute, University College London, London, United Kingdom

## Abstract

**Importance:**

The COVID-19 pandemic resulted in delayed access to medical care. Restrictions to health care specialists, staff shortages, and fear of SARS-CoV-2 infection led to interruptions in routine care, such as early melanoma detection; however, premature mortality and economic burden associated with this postponement have not been studied yet.

**Objective:**

To determine the premature mortality and economic costs associated with suspended melanoma screenings during COVID-19 pandemic lockdowns by estimating the total burden of delayed melanoma diagnoses for Europe.

**Design, Setting, and Participants:**

This multicenter economic evaluation used population-based data from patients aged at least 18 years with invasive primary cutaneous melanomas stages I to IV according to the American Joint Committee on Cancer (AJCC) seventh and eighth editions, including melanomas of unknown primary (T0). Data were collected from January 2017 to December 2021 in Switzerland and from January 2019 to December 2021 in Hungary. Data were used to develop an estimation of melanoma upstaging rates in AJCC stages, which was verified with peripandemic data. Years of life lost (YLL) were calculated and were, together with cost data, used for financial estimations. The total financial burden was assessed through direct and indirect treatment costs. Models were building using data from 50 072 patients aged 18 years and older with invasive primary cutaneous melanomas stages I to IV according to the AJCC seventh and eighth edition, including melanomas of unknown primary (T0) from 2 European tertiary centers. Data from European cancer registries included patient-based direct and indirect cost data, country-level economic indicators, melanoma incidence, and population rates per country. Data were analyzed from July 2021 to September 2022.

**Exposure:**

COVID-19 lockdown-related delay of melanoma detection and consecutive public health and economic burden. As lockdown restrictions varied by country, *lockdown scenario* was defined as elimination of routine medical examinations and severely restricted access to follow-up examinations for at least 4 weeks.

**Main Outcomes and Measures:**

Primary outcomes were the total burden of a delay in melanoma diagnosis during COVID-19 lockdown periods, measured using the direct (in US$) and indirect (calculated as YLL plus years lost due to disability [YLD] and disability-adjusted life-years [DALYs]) costs for Europe. Secondary outcomes included estimation of upstaging rate, estimated YLD, YLL, and DALY for each European country, absolute direct and indirect treatment costs per European country, proportion of the relative direct and indirect treatment costs for the countries, and European health expenditure.

**Results:**

There were an estimated 111 464 (range, 52 454-295 051) YLL due to pandemic-associated delay in melanoma diagnosis in Europe, and estimated total additional costs were $7.65 (range, $3.60 to $20.25) billion. Indirect treatment costs were the main cost driver, accounting for 94.5% of total costs. Estimates for YLD in Europe resulted in 15 360 years for the 17% upstaging model, ranging from 7228 years (8% upstaging model) to 40 660 years (45% upstaging model). Together, YLL and YLD constitute the overall disease burden, ranging from 59 682 DALYs (8% upstaging model) to 335 711 DALYs (45% upstaging model), with 126 824 DALYs for the real-world 17% scenario.

**Conclusions and Relevance:**

This economic analysis emphasizes the importance of continuing secondary skin cancer prevention measures during pandemics. Beyond the personal outcomes of a delayed melanoma diagnosis, the additional economic and public health consequences are underscored, emphasizing the need to include indirect economic costs in future decision-making processes. These estimates on DALYs and the associated financial losses complement previous studies highlighting the cost-effectiveness of screening for melanoma.

## Key Points

**Question:**

What were the consequences associated with delays in melanoma screenings during pandemic-related lockdowns, in terms of years of life lost and economic costs?

**Findings:**

This economic evaluation using population data from 31 European countries estimated that delayed melanoma diagnoses due to pandemic lockdowns were associated with 111 464 years of life lost, incurring additional costs of US$7.65 billion, signifying a significant burden on European health care.

**Meaning:**

These findings highlight the need for sustained prevention strategies and the substantial public health and economic implications of delayed melanoma diagnosis during pandemics.

## Teaser

This economic evaluation assesses the premature mortality and economic costs associated with suspended melanoma screenings during COVID-19 pandemic lockdowns by estimating the total burden of delayed melanoma diagnoses for Europe.

## Introduction

According to the global cancer observatory, melanoma was the seventh most frequent cancer entity in Europe in 2020.^1^ The median monthly growth rate of cutaneous melanomas varies between 0.1 mm for superficial spreading melanomas and 0.5 mm for nodular melanomas, while rapid growth is associated with tumor thickness, being an older man, and having fewer than 50 nevi.^2^ The potential to metastasize is highly variable depending on the growth rate, ranging between 1 month to more than 5 years.^3^

The COVID-19 pandemic and corresponding lockdowns have posed a significant challenge to health care access, including for elective skin cancer screenings. This limitation on secondary cancer prevention strategies raises concerns about a postlockdown increase in cancer diagnoses, including melanoma. According to a modeling estimation based on US outpatient health record reviews covering 4.7 million patients, the mean pandemic-related diagnostic delay of cutaneous melanomas was 1.8 months.^4^

Every disease carries associated economic costs. While direct costs are treatment-related medical costs, indirect costs are economic losses resulting from reduced productivity. Indirect treatment costs can be calculated using disability-adjusted life-years (DALYs), with 1 DALY equal to 1 year of healthy life lost. DALYs are calculated as the sum of years of life lost (YLL) and years lost due to disability (YLD) for patients living with a health condition.^5^ A previous Australian modeling study of direct health care costs estimated an increase in these costs for treating the T1 melanoma population diagnosed in 2020 to be US$6.8 million (A$9.1 million) higher following a 3-month delay and US$27.3 million (A$36.4 million) higher following a 6-month delay due to the COVID-19 pandemic.^6^ Another national population-based modeling study from England evaluated excess cancer deaths due to diagnostic delay during the COVID-19 pandemic across 4 major cancer types (breast, lung, bowel, and esophageal cancer) and estimated additional productivity losses of US$142.8 million (£103.8 million) in the next 5 years.^7^ In this study, we aimed to better elucidate potential societal and economic bearings of a pandemic-related delay in melanoma diagnosis by investigating the total burden for Europe using YYL as well as direct and indirect costs.

## Methods

This economic evaluation was approved by the Northwest and Central Switzerland Ethics Committee with a waiver of informed consent because this research does not fall under the Human Research Act. This report meets the criteria for each item in the Consolidated Health Economic Evaluation Reporting Standards (CHEERS) reporting guideline .

### Input Data

**Figure 1.**
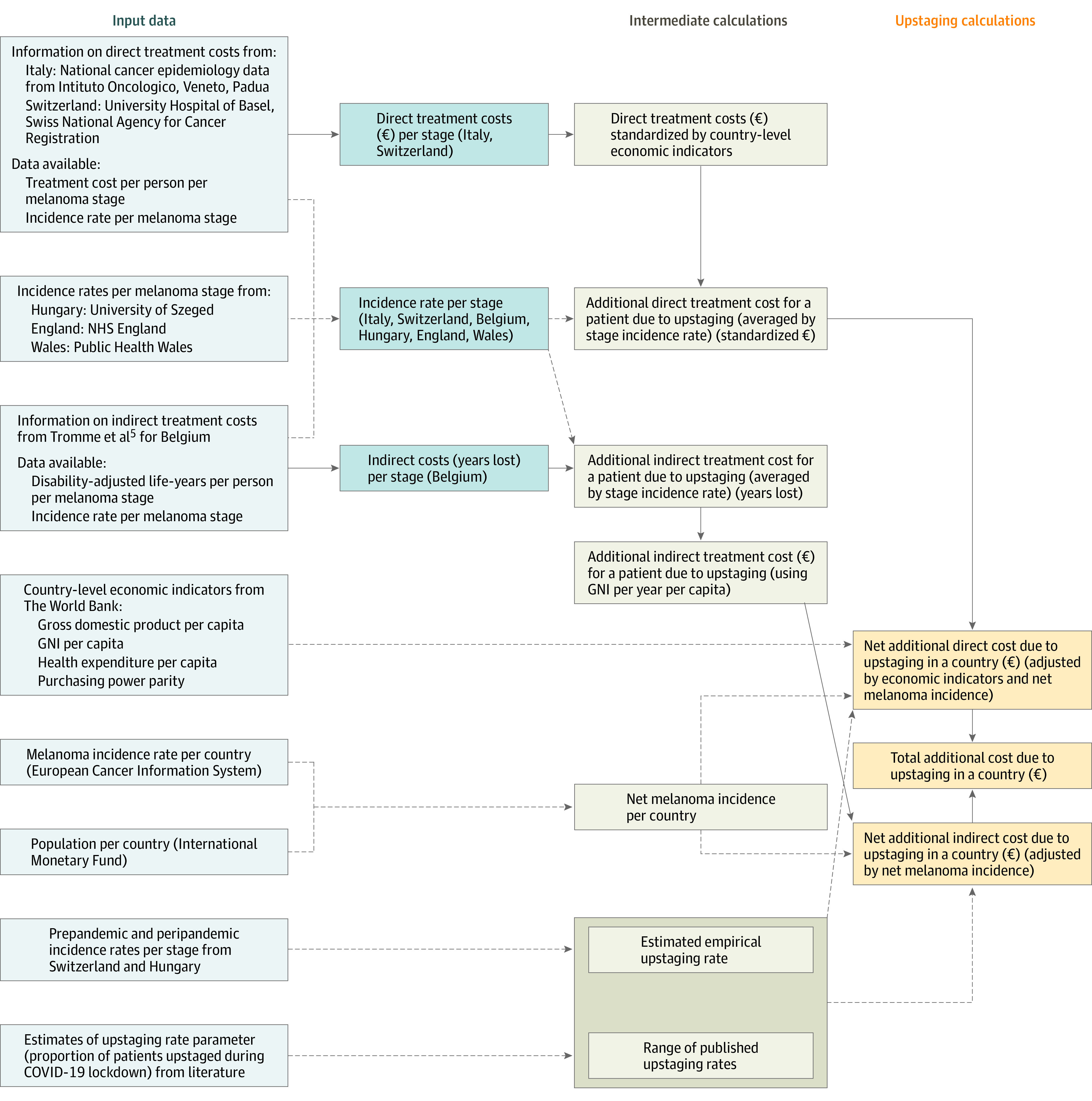
Flowchart of the Model Generation Process Upstaging-induced costs estimated for melanoma include direct, indirect, and total treatment costs and are based on registry data from Italy, Switzerland, and Belgium. Information on direct treatment costs (eTable 2 in Supplement 1) was available from University Hospital Basel, Switzerland (cost values from unpublished data, and number of patients from the National Agency for Cancer Registration) and the Veneto Region, Italy (cost values from Buja et al,^8^ and number of patients from unpublished data). For the calculation of disease burden, previously published data from the Belgian cancer registry were used.^5^ Estimation of upstaging rates using prepandemic and peripandemic incidence data from Switzerland and Hungary is shown at the bottom. A range of upstaging rate parameter values were used in this study based on the estimates provided by some recent publications.^6,8^ Light blue boxes are input data sources; darker blue boxes, input data extracted from those sources grouped into data types; beige boxes, intermediate calculation steps; orange boxes, final calculated values of upstaging costs. Arrows indicate flow of data into different phases of the modeling process; arrows transmitting cost information are solid, arrows transmitting other information, such as rates or parameters, are dashed. GNI indicates gross national income; NHS, National Health Service.

For this international, population-based modeling approach, we included patient-based direct and indirect cost data, country-level economic indicators, cutaneous melanoma incidence rates per country, and population per country numbers (Figure 1). We obtained databases on a total of 50 072 patients from sources outlined in eTables 1 to 3 in Supplement 1. Our input data included patients with melanoma with American Joint Committee on Cancer (AJCC) stage information for the direct treatment costs calculation from the University Hospital Basel, Switzerland, from 2017 to 2020, and the Veneto Region in Italy from 2016.^8^ Further, we included data on melanoma incidence from the Swiss National Cancer Registry from 2013 to 2015, from Hungary from 2019, from National Health Service England from 2015 to 2017,^9^ and from Public Health Wales from 2016 to 2017.^10^ Indirect treatment costs and melanoma numbers were collected from the Belgian Cancer Registry from 2009 to 2011.^5^ We included patients aged 18 years and older with invasive primary cutaneous melanomas stages I to IV, according to the AJCC seventh and eighth editions, including melanomas of unknown primary (T0).^11,12,13^ Additional information is provided in eTable 1 and eTable 4 in Supplement 1.

### Definition of Lockdown

As a reaction to the COVID-19 pandemic, restrictions concerning many areas of daily life were installed by many countries, beginning in March 2020. For example, access to medical institutions was strictly limited to the treatment of emergency situations, such as myocardial infarction, mainly to create capacity to care for the sudden large number of patients with COVID-19. The types of restrictions and their duration varied greatly from country to country. In our study, we refer to the following lockdown scenario: elimination of routine medical examinations and severely restricted access to follow-up examinations for at least 4 weeks.

### Definition of Upstaging

The definition of upstaging is reclassification of the stage of cancer to a higher, more advanced stage based on clinicopathological characteristics according to previous publications.^14^ We used lockdown-related postponements and cancellations resulting in delay of melanoma diagnosis as reasons for upstaging.

### Statistical Analysis

#### Incidence Rate Estimation

**Figure 2.**
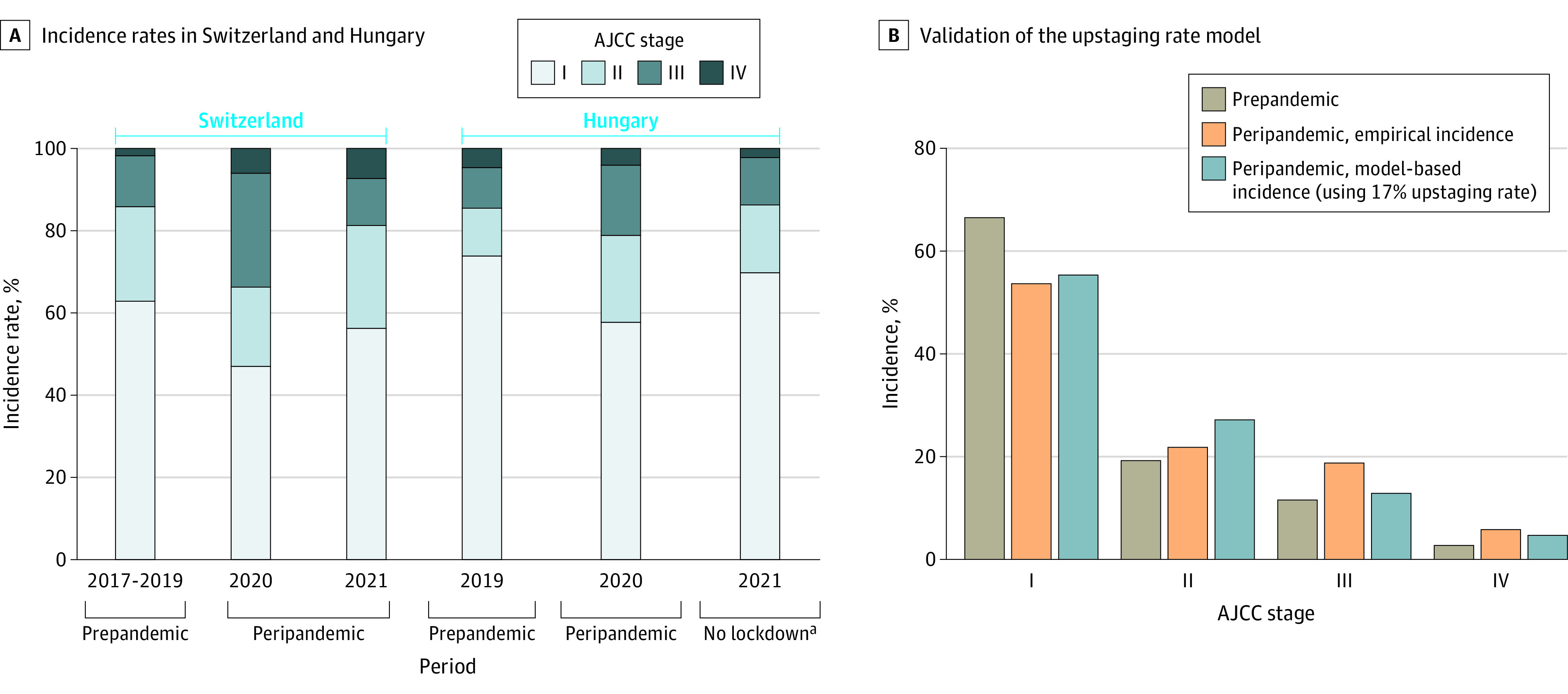
Upstaging Scenarios and Rates Associated With COVID-19 Lockdown-Related Delayed Melanoma Diagnosis A, American Joint Committee on Cancer (AJCC) upstaging rates were estimated for the perilockdown years and compared with the prepandemic incidence rates. B, AJCC stage-based incidence rates for before and during the COVID-19 pandemic. Proportions were combined from the Swiss (2020 and 2021) and Hungarian (2020) cohorts. The empirical peripandemic proportions are compared against the incidence rates estimated by the upstaging rate–based model, applied on the prepandemic incidence proportions, with the estimated upstaging rate of 17% (estimated by comparing the peripandemic and prepandemic empirical incidence rates from the 2 cohorts). ^a^Hungary did not experience lockdown during 2021, so its data have been excluded in subsequent analyses.

**Figure 3.**
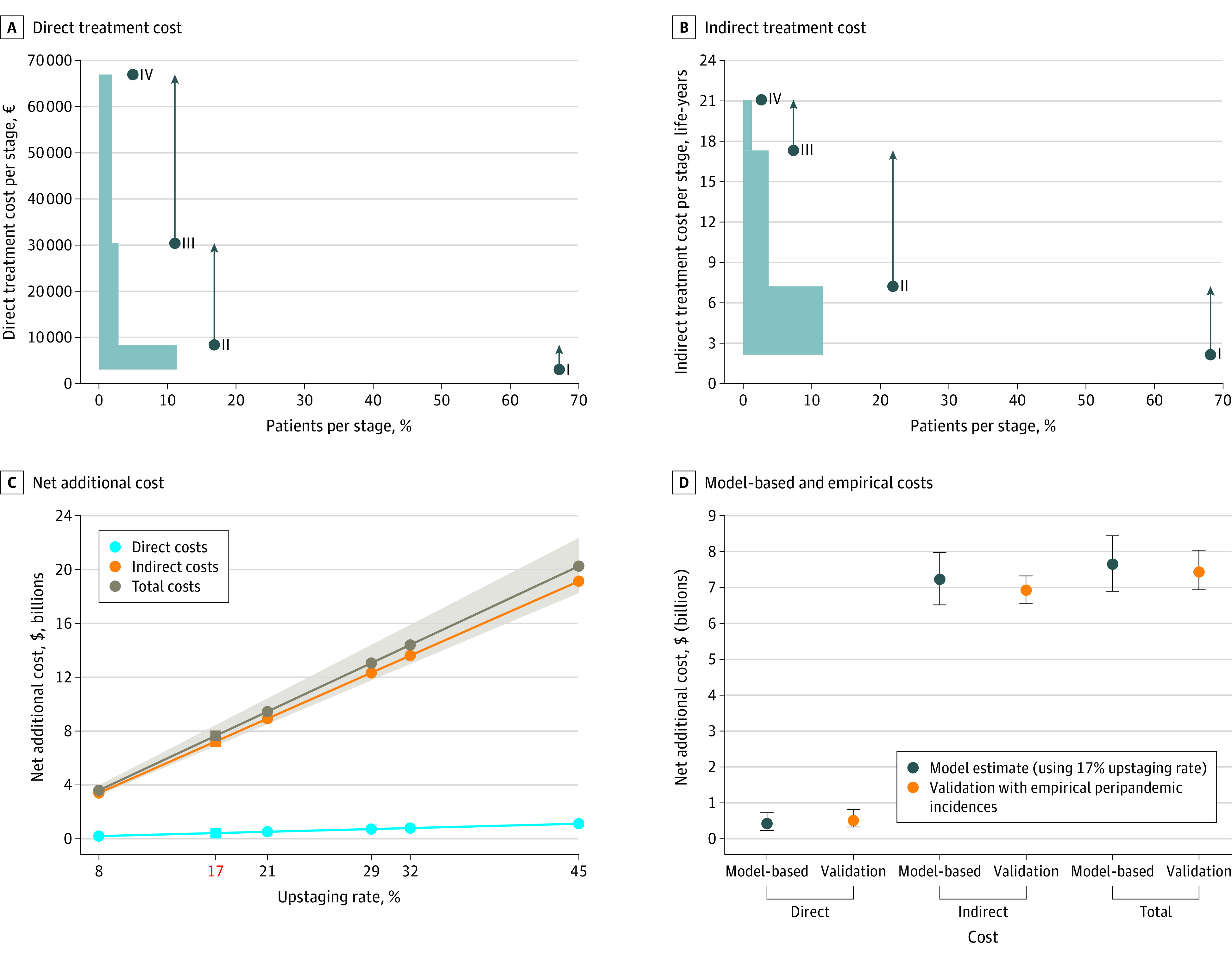
Estimation of Treatment Costs for Europe Associated With COVID-19 Lockdown-Related Delayed Melanoma Diagnosis A, Additional direct treatment costs were estimated for a 17% upstaging rate based on incidence rates from the Italian registry.^15^ Points correspond to the 4 American Joint Committee on Cancer (AJCC) stages, their x-axis values represent the proportion of individuals affected for this tumor stage (in percentage), and their y-axis values represent the mean treatment cost at this tumor stage (eTable 1 in Supplement 1). The increase in costs when a person moves up one stage is shown by the arrow. The increases in treatment costs from each stage are represented as rectangular shading, and the width of the rectangle is the proportion of people at a tumor stage who are upstaged and is calculated by multiplying the proportion of individuals affected per tumor stage (x-axis value of the point) with the 17% upstaging rate (empirical data). The height of the rectangular shading is the estimated mean cost increase due to upstaging, shown by the arrow (y-axis). The rise in costs for each stage is therefore the area of the column shading, its width (proportion of people upstaged) × height (cost rise). The total area of the rectangular shading represents the aggregate rise in direct costs due to upstaging. B, Additional indirect treatment costs were estimated for a 17% upstaging rate based on the Belgian registry^5^ (eTable 2 in Supplement 1). The increases in indirect costs for each stage (rectangular shading) are generated from multiplying the width ie, the proportion of individuals affected per tumor stage (x-axis, in percentage) with the height, ie, increase in disability-adjusted life-years per stage. C, This model includes 5 different upstaging rate scenarios, which have been described in literature.^6,8^ The shading represents the 95% CI of the total costs. The 17% upstaging rate (estimated from empirical data) is shown on the x-axis in red, and its corresponding values are highlighted as squares. The cost model equations are: direct costs (in billions of US$) = 0.0248 × upstaging rate (percentage); indirect costs = 0.4251 × rate; total costs = 0.4499 × rate. D, Estimates of the rise in direct, indirect, and total costs for Europe, obtained from the linear cost model shown in C, for the 17% upstaging rate estimated from empirical data, are shown in blue. We checked the validity of these estimates of additional costs by directly using the prepandemic and peripandemic incidence rates for Switzerland and Hungary (which were also used in estimating the upstaging rate) in conjunction with the stagewise mean costs, to calculate the extent of cost rise during the pandemic without any model assumptions. These estimates are shown in orange. Error bars indicate 95% CIs.

Numbers of patients at each melanoma stage were taken from cancer registry databases (eTables 1-3 in Supplement 1), and their proportions were taken as estimates of incidence rates of each melanoma stage (Figure 2A). These proportions were combined with per-stage estimates of indirect costs (Belgian data) (eTable 2 in Supplement 1) or direct costs (Italian and Swiss data) (eTable 1 in Supplement 1) to perform an upstaging calculation (Figure 3A and B) to obtain an estimate of additional cost due to upstaging. Calculation examples of the estimation of additional indirect costs (as YLL) due to upstaging additional and of direct costs due to upstaging are outlined in the eMethods and eTable 7 in Supplement 1. All calculations were performed using R statistical software version 4.2.0 (R Project for Statistical Computing), with packages tidyverse and ggplot2.

#### Model Development

Figure 1 and Figure 3A and B explain calculation of additional costs incurred due to upstaging. For estimating the additional indirect cost due to upstaging in life-years for a specific upstaging rate (*u*), we used stage-wise indirect costs calculated as DALYs using Belgian data and incidence rates from 6 registries. By doing the calculations then using the mean of the estimates, we approximated the use of a mean incidence rate across the European Union. DALYs, in our study, encompass both the health-related restrictions and the estimated financial costs of delayed melanoma diagnosis. In addressing the concept of discounting DALYs, we acknowledge the complexity and debate surrounding this issue. Discounting future earnings against economic growth is a nuanced process, and assumptions regarding growth rates and discount rates can significantly impact cost-effectiveness analyses. However, for pragmatic reasons and to account for inherent uncertainties, we have assumed, as a starting point, that these forces may neutralize each other. Using the country’s gross national income (GNI) per capita as a basis implicitly considers labor market participation. In economies with weaker labor metrics, the GNI per capita is understandably lower, indirectly reflecting the economic productivity and participation rates.

Simultaneously, there are ethical concerns of discounting DALYs. The process raises critical questions about fairness across generations, the potential undervaluation of long-term health benefits, and its impact on vulnerable groups, including individuals facing economic or social disadvantage, living in certain geographical locations, very young and very old individuals, women and girls, ethnic and cultural minority populations, persons with disabilities, and migrants and refugees, all of whom often experience higher DALYs due to social factors, like limited health care access, poor living conditions, discrimination, and specific health risks related to their situations. Moreover, discounting might conflict with societal values by potentially undervaluing future health outcomes. Given these considerations, and to avoid undervaluing interventions with long-term benefits, we have chosen a cautious approach by not discounting our DALYs. While DALYs do not explicitly include labor force participation metrics, they indirectly consider the impact of health conditions on productivity. Severe health conditions or disabilities, as captured by DALYs, affect an individual’s ability to participate in the labor force, thereby influencing their productivity and economic contributions. Therefore, our approach seeks to balance the practicality of economic evaluation with the ethical considerations surrounding the use of DALYs. By using this approach, we aim to provide a comprehensive and thoughtful analysis that respects the complexity of this topic. We believe that this balanced approach enhances the robustness and relevance of our findings in the context of delayed melanoma diagnosis during the COVID-19 pandemic.

In addition to our health-related data from Belgium, a 2022 report from the US using data from 1990 to 2017^16^ indicated that melanoma-related DALYs have not changed significantly and do not vary substantially between the US and Belgium. Hence, we consider the Belgian data still relevant for our analysis.

To estimate indirect financial costs of delayed melanoma diagnosis, we converted these estimates from life-years per person (DALY units) to euro by multiplying life-years per person by GNI per capita per country.^17^ Each DALY lost thus creates a financial cost equivalent to the country’s GNI per capita. Note that unlike gross domestic product (GDP) per capita or mean wages, GNI per capita provides a comprehensive account for residents’ incomes. We then multiplied these numbers by net melanoma incidence for each country to calculate net additional indirect cost due to upstaging (in euro), based on previous models.^17^ While future earnings should in principle be discounted, assuming economic growth and increasing wages, there is also a counterforce. Direct costs from each source were converted to direct costs (in euro) for each target country by using GDP, health expenditure (HE), and purchasing power parity, resulting in a standardized cost value for each country, based on previous models.^17^

The cost value for a country can be obtained 6 different ways, using each of the 2 source country’s costs (Italy or Switzerland) and any of the 3 economic indicators (GDP, HE, or purchasing power parity). For each set of cost values per melanoma stage, an estimate of the additional direct cost due to upstaging was calculated following the modeling procedure. We calculated the mean of these estimates and multiplied by the net melanoma incidence for each country to calculate the net additional direct cost due to upstaging in a country (in euro). All calculated monetary costs were finally reported in US dollars, converting all euro numbers to US dollars using a conversion rate of €1 to $1.11, the mean exchange value during the year 2020.

Probabilistic sensitivity analysis was used to represent the modeling uncertainty and produce 95% CIs. Detailed methods are described in the eMethods, eTable 7, and eTable 8 in Supplement 1. Population sizes and economic indicators are outlined in eTable 5 in Supplement 1. A similar approach was used across primary cutaneous melanoma tumor thickness categories (ie, AJCC T categories).

#### Upstaging Scenarios in Europe

Certain countries, including Germany and Italy, have experienced a decrease in melanoma diagnoses from March to May 2020 (Germany) and May to July 2020 (Italy),^15,18^ suggesting an impending increase in advanced-stage melanoma. Detailed upstaging rate estimates for melanoma were available from 2 studies based on AJCC T categories (not considering N and M stages).^6,19^ The model by Degeling et al^6^ was developed using Australian melanoma registry data and considering tumor thickness shifts from primary cutaneous melanoma T1 to T2 category (AJCC seventh edition), with an estimated 8% upstaging rate after a 3-month delay and a 32% upstaging rate after a 6-month delay. Tejera-Vaquerizo and colleagues^19^ estimated a 21% upstaging rate in the 1-month delay group, 29% upstaging rate in the 2-month delay group, and 45% upstaging rate in the 3-month delay group using a Spanish data set from an Oncology Department (AJCC eighth edition) and shifts from primary cutaneous melanoma T1 to T4 category. As the 2 studies from the literature^6,19^ provided the background for our comparisons, we used their different upstaging rates (based on AJCC T categories) in Figure 3C. We assumed that upstaging rates are equal across all AJCC stages and developed a linear model (eTable 8 in Supplement 1). We confirmed our assumption by using our health registry data, in which we detected a 17% upstaging rate for AJCC stages. Cost increases under this model are proportional to the upstaging rate parameter.

#### Estimation of Upstaging Rates

From the Swiss and Hungarian data displayed in Figure 2A, we built a statistical model to estimate the upstaging rate parameter from these prepandemic and peripandemic incidence rates (eTable 8 in Supplement 1). The estimated upstaging rates (based on AJCC, seventh edition) were 18.0% in 2020 and 15.8% in 2021 (eTable 6 in Supplement 1). Pandemic incidence rates from the University Hospital in Szeged, Hungary, in 2020 were compared with incidences before the pandemic in 2019, when the estimated rate was 16.6% (eTable 6 in Supplement 1). We calculated the mean, resulting in an upstaging rate of 16.8% (rounded to 17%), which was used for subsequent calculations. Finally, the association between lockdowns and cancer upstaging rates was verified by using the 2021 data from Hungary, as no lockdowns were introduced in 2021,^20^ resulting in a 2021 upstaging rate of only 0.4% (eTable 6 in Supplement 1).

We confirmed the interchangeability between the AJCC seventh^11^ and eighth editions^12,13^ (AJCC stage and T categories) (eTable 4 in Supplement 1). The variation in estimated rates was not more than 0.5 percentage points (eTable 6 in Supplement 1). Since the published DALY calculations are based on the AJCC seventh edition,^5^ we used AJCC seventh edition^11^ staging for data used in the primary modeling.

#### Validation of the Use and Estimation of Upstaging Rates

We included 405 patients from the Department of Dermatology, University Hospital, Basel, Switzerland with cutaneous melanoma AJCC stage IA to IV between January 2017 and December 2021 and 477 patients from the Department of Dermatology and Allergology, University of Szeged, Szeged, Hungary, between January 2019 and December 2021. Both university hospitals offer multimodal treatment options according to the newest guidelines, providing standardized data collection and follow-up. Thus, we used these patients from both tertiary centers for recording the real melanoma costs and the upstaging estimations. These costs included the initial diagnostics and staging procedure as well as the interventions and medications based on the current guideline, follow-up examinations, and supportive care during the first follow-up year. Mucosal melanoma and melanoma in situ (stage 0) were excluded in both tertiary centers. We consider an incorporation of upstaging based on data from 2 countries into our model to extrapolate to all European countries a valid method, since it is based on 2 countries with 2 different health care systems and it covers the range of Eastern and Western European countries with high and lower incomes.

The peripandemic incidence rates estimated by the 17% upstaging rate model (applied on prepandemic incidence rates) are comparable with real peripandemic incidence rates for the 2 countries, since they fall within the 8% to 45% upstaging rates previously reported.^6,19^ Thus, an upstaging rate of 17% was used for all subsequent upstaging calculations.

We further validate the use of our model by calculating the cost rise directly by using real-life incidence numbers and cost values, without any modeling assumptions. These values and 95% CIs are compared with the cost estimates and 95% CIs from the upstaging rate-based cost model, where the upstaging rate parameter of 17% was used (eMethods in Supplement 1).

## Results

### Estimation of Disease Burden Due to Delayed Melanoma Diagnosis

**Figure 4.**
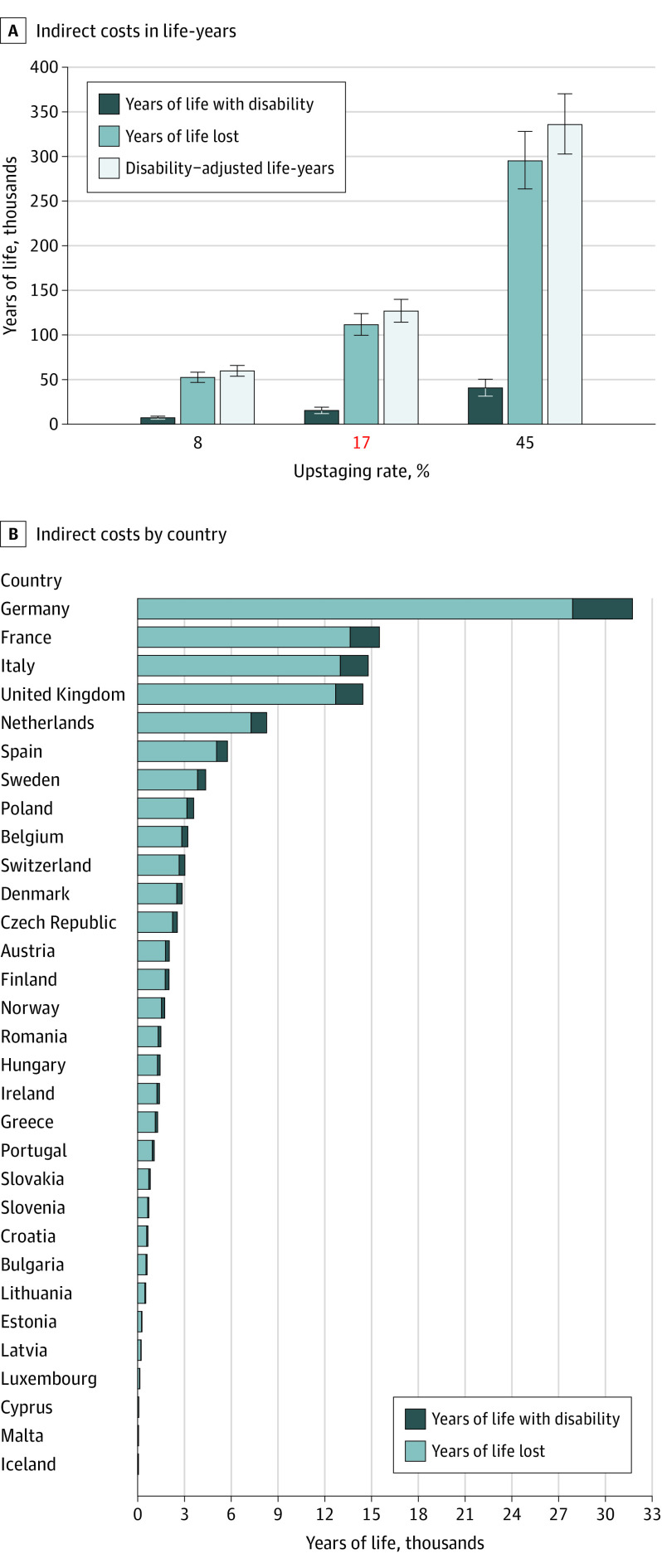
Estimated Increases of Years of Life With Disability, Years of Life Lost, and Disability-Adjusted Life-Years Associated With COVID-19 Lockdown-Related Delayed Melanoma Diagnosis A, The 17% upstaging rate (estimated from empirical data) is highlighted on the x-axis in red. The 8% and 45% upstaging rates are the minimum and maximum rates reported in the literature. Errors bars indicate 95% CIs. B, Data are estimated under the 17% upstaging model.

We estimated 111 464 additional YLL associated COVID-19 lockdown-related delayed melanoma diagnosis in Europe for the 17% upstaging model, ranging from of 52 454 (8% upstaging scenario) to 295 051 YLL (45% upstaging scenario) (Figure 4A). Estimates for YLD in Europe resulted in 15 360 years for the 17% upstaging model, ranging from 7228 years (8% upstaging model) to 40 660 years (45% upstaging model). Together, YLL and YLD constitute the overall disease burden, ranging from 59 682 DALYs (8% upstaging model) to 335 711 DALYs (45% upstaging model), with 126 824 DALYs for the real-world 17% scenario (Figure 4A).

To understand the bearing of this disease burden for Europe, YLD and YLL are displayed for each of the countries separately (with Figure 4B showing the 17% real-life scenario). In line with previous data,^5^ the contribution of YLDs to DALYs was relatively small (mean, 12.5%) yet important nonetheless.^6^

### Cost Estimation Due to Delayed Melanoma Diagnosis

To calculate costs, we estimated additional costs based on our model by calculating the extent of cost increase using stagewise incidence rates. All calculations were based on a specific value of the upstaging rate parameter (*u*), ranging from 8% to 45% as outlined in Figure 3C. The cost increase was proportional to the upstaging rate parameter (eMethods in Supplement 1). The estimates (direct, indirect, and total) for each of the 31 European countries were added to obtain aggregate costs for Europe as a whole.

The lockdown-related delay in melanoma care was associated with upstaging and increasing financial costs for Europe. This cost increase results from an increase of direct (Figure 3A) and indirect (Figure 3B) costs, which together sum to total costs (Figure 3C).

Melanomas are differentiated into 4 stages in the AJCC seventh edition, I through IV.^11^ As outlined in Figure 3A and B and eTables 1 to 4 in Supplement 1, most melanomas were detected at an early stage (eg, for stage I, 66%; stage II, 24%; stage III, 7%; stage IV, 3%; according to National Health Service England), requiring few invasive diagnostic examinations and rarely treatment beyond surgery.^21^ Higher stages require more significant diagnostic examinations, patient visits, and expensive treatments (Figure 3A and B).^21^ Mean (SD) treatment costs ranged from of €3049 (€7826) for stage I to €66 950 (€42 977) for stage IV in the Italian cohort.^8^ The excess costs generated from upstaged lesions are displayed in Figure 3A (direct costs) and Figure 3B (indirect costs) and eTable 5 in Supplement 1. Mean direct and indirect costs in DALYs increased with each stage increase.

Finally, total additional costs were calculated (Figure 3C; eTable 5 in Supplement 1). The range of the upstaging rate spans from the most conservative 8% upstaging rate (additional direct costs, $0.20 [95% CI, $0.11-$0.34] billion; additional indirect costs, $3.40 [95% CI, $3.07-$3.75] billion; total additional costs, $3.60 [95% CI, $3.24-$3.97] billion) up to the more aggressive 45% upstaging rate (total additional costs, $20.25 [95% CI, $18.25-$22.34] billion; including $1.12 [95% CI, $0.60-$1.92] billion in direct costs and $19.13 [95% CI, $17.25-$21.10] billion in indirect costs). With the actual 17% upstaging rate, our model estimated the total additional costs to be $7.65 (95% CI, $6.89-$8.44) billion, including $0.42 (95% CI, $0.23-$0.72) billion in direct costs and $7.23 (95% CI, $6.52-$7.97) billion in indirect costs.

To validate our model, we used real-world stagewise incidence rates before and during the pandemic from 2 countries in conjunction with the stagewise costs to calculate the extent of cost increase during the pandemic without any model assumptions. The estimated mean (SD) additional costs were $0.51 (95% CI, $0.33-$0.82) billion in direct costs, $6.93 (95% CI, $6.55-$7.32) billion in indirect costs, and $7.43 (95% CI, $6.94-$8.04) billion in total costs (Figure 3D). All these values estimated without model assumptions were close to our model estimates with a 17% upstaging rate and fall within each other’s 95% CIs (Figure 3D), thereby validating our model.

In addition to the significant increase of total costs, these data shed light on the importance of indirect costs, which were approximately 94.5% of total costs. These indirect costs include sick leave–associated costs, productivity losses due to morbidity, and premature mortality.

### Contribution of the Upstage-Driven Additional Economic Costs to European Health Care Expenses

**Figure 5.**
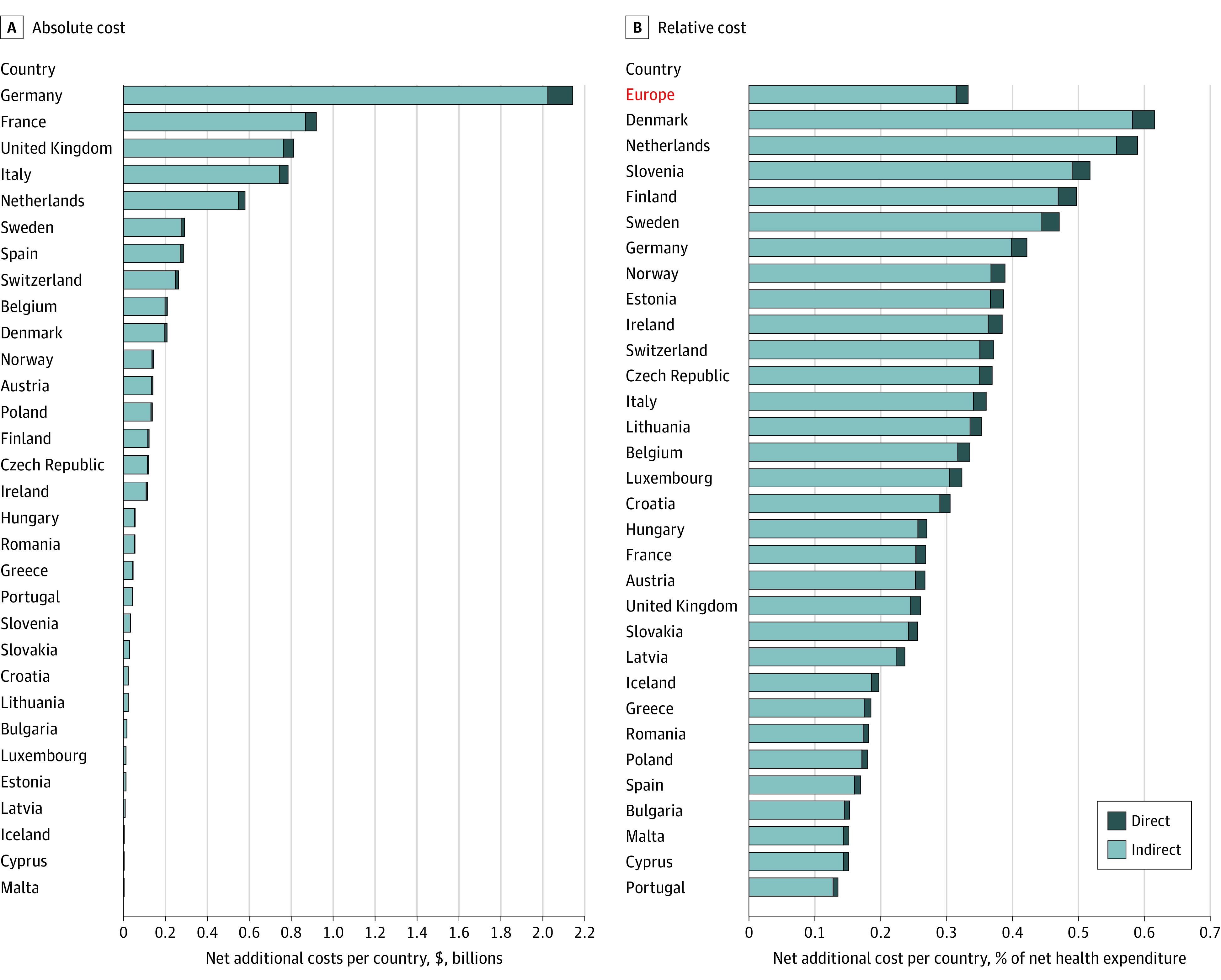
Upstaging-Driven Additional Economic Costs Associated With COVID-19 Lockdown-Related Delayed Melanoma Diagnosis A, Absolute direct and indirect treatment costs per European country are presented for the 17% upstaging rate. B, Relative direct and indirect treatment costs are displayed in relation to the country’s health expenditure using the 17% upstaging rate. The aggregated estimate for all of Europe is highlighted on the y-axis in red.

Melanoma incidences in Europe show a broad variation, from 7.9 melanomas per 100 000 inhabitants per year in Romania to as high as 50.3 melanomas per 100 000 inhabitants per year in Denmark, according to the European Cancer Information System. Figure 5 presents cost calculations in which the 17% upstaging rate has been applied to 31 European countries, displaying the estimated increase of total treatment costs for all European countries (Figure 5A), ranging from $3.08 (95% CI, $2.78-$3.40) million in Malta to $2.14 (95% CI, $1.93-$2.36) billion in Germany. For Europe, this 17% upstaging model estimates total additional costs of $7.65 (range, $3.60 to $20.25) billion. For each country, the estimated net additional direct and indirect costs due to upstaging were added (Figure 5; eTable 5 in Supplement 1). Since absolute numbers of patients with melanoma tend to be larger in a bigger country (even if incidences differ), the upstaging-related cost increases are compared with a benchmark value for each European country. We chose national HE as a benchmark and estimated the country-specific additional economic costs (Figure 5A; eTable 5 in Supplement 1) as fraction of the net HE (Figure 5B) anticipating a mean increase of 0.33% (95% CI, 0.30%-0.37%) of the current European HE.

## Discussion

In this economic evaluation, the COVID-19 pandemic lockdown offered a unique chance to assess the social and economic consequences associated with delayed melanoma diagnosis in Europe, focusing on all invasive stages. Our data shed light on how the use of lockdowns as a mechanism to control SARS-CoV-2 infection rates was associated with the progression of other medical conditions.

Our upstaging model allowed us to calculate the additional economic costs associated with delay in melanoma diagnosis. These estimates complement previous studies highlighting the cost-effectiveness of screening for melanoma.^22^ Our 17% upstaging scenario was equivalent to 0.33% of the European HE. Indirect treatment costs accounted for 94.5% of total costs in our model, highlighting the hidden financial implications of delaying cancer diagnosis, in accordance with a similar study in the UK from Gheorghe et al,^7^ which estimated significant losses in quality-adjusted life-years and increased economic burdens due to excess cancer-related deaths, particularly for major cancers, like breast, colorectal, and lung cancer, using a population-based model. Their approach, using human capital metrics, revealed that the economic outcomes associated with delayed cancer care, on a per-capita basis, exceeded those of COVID-19 deaths.^7^ This underlines the critical need for effective cancer care even during pandemic-related health care disruptions. Comparing the increase of direct costs associated with diagnostic delay with the model from Australia, where Degeling et al^6^ estimated considerable excess costs over 5 years due to stage progression in cancers, including melanoma, following diagnostic delays, we come to very similar findings. In our model, direct costs increased by 0.018%, while Degeling et al^6^ described an increase of 0.0041% of the total HE after a 3-month delay and 0.017% of the total HE after a 6-month delay.^23^ This was echoed by international studies that highlighted the exacerbated financial challenges faced by patients with cancer during the pandemic, particularly due to unemployment and economic recession, leading to increased out-of-pocket expenses and potentially foregoing essential treatments.^24,25^ These studies collectively emphasize the multifaceted impact of delayed cancer diagnoses, resonating with our findings on melanoma in Europe and reinforcing the imperative of maintaining robust cancer screening and care during global health crises.

Lockdown-associated delayed melanoma diagnoses resulted in a significant decline in incidence during the first lockdown period and a rise of newly diagnosed melanoma in the postlockdown phase.^15,18^ A 2022 population-based nationwide pathology registry analysis found a small shift toward unfavorable pT stages during the first lockdown compared with the pre–COVID-19 period, consistent with our upstaging model.^26^ Several studies on other cancers have reported an alarming increase in later-stage cancers in line with our real-world data, associated with reduced survival of these patients.^27,28^ Thus, concerns about an impending COVID-19–associated cancer pandemic are increasing.^29^

Various countermeasures are conceivable to mitigate the consequences of lockdowns; for example, considering increased utilization of virtual health care services for melanoma screening, as well as heightened patient education regarding warning signs of melanoma, might be worthwhile. Artificial intelligence–based digital health applications are also likely to be increasingly used in the future.^30^

Our methods can be applied more broadly and contribute to deepening the understanding of the implications of delayed diagnosis of other conditions wherever reliable incidence and cost data are available.^6^ Our approach facilitates modeling of the implications in individual countries that may differ in parameters, such as lockdown duration, population size, economic factors, and melanoma incidence.

YLLs due to fatal outcomes of non–COVID-19 conditions are a robust measure for secondary consequences of COVID-19.^31^ The estimated 52 454 YLLs to 295 051 YLLs imply a loss of 617 to 3469 full lives of 81.3 years,^32^ the mean life expectancy in our sample. These numbers account for YLL rates of 6.7 to 37.7 YLLs per 100 000 population in our model. Comparing these with 2021 estimates by Pifarré et al^31^ of YLL due to COVID-19 (15.7 for males and 15.1 for females, using data from 81 countries) is highly concerning, given that our YLL estimates relate to melanoma only.

Furthermore, in 2016, melanoma accounted for 0.065% of the total global indirect costs measured as DALYs.^32^ While our model cannot be extrapolated to all other diseases, it seems that the estimated $7.7 billion is only the tip of the iceberg, so the total burden associated with to pandemic-related delayed diagnosis and treatment may be unprecedented, both medically and economically.

### Limitations

The results need to be interpreted with caution due to several modeling limitations, including the impact of simplifying assumptions on estimations based on the extrapolation of results from modeling validation in the real-world setting limited to 2 sites to the rest of Europe. Lockdown definitions varied among different countries regarding duration and specific measure. In the context of a wide range of COVID-19 mitigation measures, combined with a wide range of country sizes, an uncertainty of the upstaging model exists, so the final cost implications may differ from our estimates. In addition, the diversity of European health care systems with differences in treatment access and disease prognosis influence total costs. Furthermore, the estimation of true health care costs is more complex than our models, as mental health is not yet reflected in DALYs. While indirect costs can be estimated from DALYs, there are several other methods that can be used to estimate indirect costs, which results in a limitation of using the DALYs. DALYs do not directly include labor force participation but account for how diseases or disabilities affect life expectancy and quality of life, which can indirectly impact someone’s ability to work. In our analysis, we opted for DALYs combined with GNI per capita over the human capital approach (HCA) to estimate indirect costs. This decision was driven by our intent to capture a more comprehensive picture of outcomes associated with delayed melanoma diagnosis, one that extends beyond mere economic productivity losses. While HCA predominantly quantifies productivity in monetary terms, our approach with DALYs allows for a broader assessment of health impacts, including quality of life and disability factors. This method aligns with our study’s goal to evaluate the multifaceted consequences associated with health care delays, balancing economic assessments with a wider health impact perspective. However, we recognize that this choice might not fully align with traditional economic evaluations focused solely on direct economic outputs, which is a considered limitation of our approach. We did not capture the effects of morbidity and mortality due to COVID-19 in the cost models, resulting in patients with melanoma being considered solely and not also as patients with COVID-19. We also did not consider data on melanoma in situ, as these figures were not available, so our method potentially underestimates the values and associations we report. We excluded mucosal and uveal melanoma due to different TNM and AJCC classification. While we used linear rise assumption as a parsimonious, pragmatic approach for our model, actual melanoma tumor biology is subject to complex, interindividual factors influencing the process of stage progression. Furthermore, melanoma treatment has rapidly evolved over the last several years, with improved patient outcomes but higher treatment costs, which may not have been fully represented in our source data. Additionally, we provide a large range for cost increase estimates to mitigate the risk of inaccuracy in extrapolating these estimates to other countries.

## Conclusions

Our economic evaluation provides relevant medical and economic insights about the implications associated with delaying melanoma diagnosis due to suspended screening, which the global COVID-19 pandemic gave us the unique opportunity to investigate. Considering the assumptions and limitations of our methods, our estimates are meant to raise awareness about the importance of skin cancer screenings and the multidimensional costs associated with screening suspension. Thus, while we urge decision-makers to consider the implications of delaying screenings in future pandemic plans to optimize public health and limit possibly severe medical and economic sequelae of containment policy, we most importantly encourage policy-makers to further emphasize the role of preventative medicine on both a personal and economic level.

## Notes

Supplement 1. **eTable 1.** Data on Direct Treatment Costs and Prepandemic Melanoma Incidence for Switzerland and Italy **eTable 2.** Data on Indirect Treatment Costs and Prepandemic Melanoma Incidence for Belgium **eTable 3.** Data on Prepandemic Melanoma Incidence for Hungary, England, and Wales **eTable 4.** Data on Melanoma Incidence Before and During the Pandemic for Switzerland and Hungary **eTable 5.** Various Characteristics and Indicators for Each Country, Along With Estimated Direct and Indirect Costs, and Years of Life Lost **eTable 6.** Estimated Upstaging Rates for Switzerland and Hungary
eMethods.
**eTable 7.** Schematic Overview of the Calculation Process for Additional Cost Incurred Due to Upstaging **eTable 8.** Schematic Overview of the Calculation Process for Upstaging Rate
eReferences.

Supplement 2.
Data Sharing Statement
