# Supplementary material for: Health Economic Consequences Associated With COVID-19–Related Delay in Melanoma Diagnosis in Europe
Source: JAMA Netw Open. 2024 Feb 16;7(2):e2356479. doi: 10.1001/jamanetworkopen.2023.56479 (PMC10873772; doi:10.1001/jamanetworkopen.2023.56479)
Supplement: Supplement 1. — eTable 1. Data on Direct Treatment Costs and Prepandemic Melanoma Incidence for Switzerland and Italy eTable 2. Data on Indirect Treatment Costs and Prepandemic Melanoma Incidence for Belgium eTable 3. Data on Prepandemic Melanoma Incidence for Hungary, England, and Wales eTable 4. Data on Melanoma Incidence Before and During the Pandemic for Switzerland and Hungary eTable 5. Various Characteristics and Indicators for Each Country, Along With Estimated Direct and Indirect Costs, and Years of Life Lost eTable 6. Estimated Upstaging Rates for Switzerland and Hungary eMethods. eTable 7. Schematic Overview of the Calculation Process for Additional Cost Incurred Due to Upstaging eTable 8. Schematic Overview of the Calculation Process for Upstaging Rate eReferences. [file jamanetwopen-e2356479-s001.pdf]

Supplemental Online Content

Maul LV, Jamiolkowski D, Lapides RA, et al. Health economic consequences associated with COVID-19–related delay in melanoma diagnosis in Europe. *JAMA Netw Open*. 2024;7(2):e2356479. doi:10.1001/jamanetworkopen.2023.56479

eTable 1. Data on Direct Treatment Costs and Prepandemic Melanoma Incidence for Switzerland and Italy
eTable 2. Data on Indirect Treatment Costs and Prepandemic Melanoma Incidence for Belgium
eTable 3. Data on Prepandemic Melanoma Incidence for Hungary, England, and Wales
eTable 4. Data on Melanoma Incidence Before and During the Pandemic for Switzerland and Hungary
eTable 5. Various Characteristics and Indicators for Each Country, Along With Estimated Direct and Indirect Costs, and Years of Life Lost
eTable 6. Estimated Upstaging Rates for Switzerland and Hungary
eMethods.
eTable 7. Schematic Overview of the Calculation Process for Additional Cost Incurred Due to Upstaging
eTable 8. Schematic Overview of the Calculation Process for Upstaging Rate
eReferences.

This supplemental material has been provided by the authors to give readers additional information about their work.

© 2024 Maul LV et al. *JAMA Network Open.*

eTable 1: Data on direct treatment costs and pre-pandemic melanoma incidence for Switzerland and Italy

**Table 1A: Switzerland data – University Hospital Basel, 2017-2020**				
**Stage**	**Number of patients**	**Proportion**	**Cost (€)**	**Cost (converted to USD)**
I	142	62.8%	€ 7,231	$ 8,026
II	52	23.0%	€ 30,600	$ 33,966
III	28	12.4%	€ 86,877	$ 96,433
IV	4	1.8%	€ 155,959	$ 173,114
**Total**	**226**			

**Table 1B: Switzerland data – Incidence numbers from NACR, 2013-2015**		
**Stage**	**Number of patients**	**Proportion**
I	2088	84.9%
II	282	11.5%
III	30	1.2%
IV	58	2.4%
**Total**	**2458**	

Note: 659 cases were excluded from the data due to missing stage information. NACR=National Agency for Cancer Registration. The data is from 9 Swiss Cantons representing 38% of the Swiss population.

**Table 1C: Italy data – Cost values[1], Number of patients from unpublished data; 2016**				
**Stage**	**Number of patients**	**Proportion**	**Cost (€)**	**Cost (converted to USD)**
I	854	67.1%	€ 3’049	$ 3,384
II	214	16.8%	€ 8’388	$ 9,311
III	141	11.1%	€ 30’403	$ 33,747
IV	63	5.0%	€ 66’950	$ 74,315
**Total**	**1272**			

Note: 96 cases were excluded from the data due to missing stage information.

© 2024 Maul LV et al. *JAMA Network Open.*

eTable 2: Data on indirect treatment costs and pre-pandemic melanoma incidence for Belgium

Table 2: Data on indirect treatment costs and melanoma numbers for Belgium [2] , 2009-2011					
**Stage**	**Number of patients**	**Proportion**	**YLD (years)**	**YLL (years)**	**DALY (years)**
I	4041	68.2%	0.52	1.63	2.14
II	1295	21.8%	1.25	5.97	7.22
III	435	7.3%	3.09	14.23	17.32
IV	157	2.6%	1.05	20.03	21.08
**Total**	**5928**				

© 2024 Maul LV et al. *JAMA Network Open.*

eTable 3: Data on pre-pandemic melanoma incidence for Hungary, England, and Wales

**Table 3A: Hungary data, 2019, from University Hospital of Szeged**		
**Stage**	**Number of patients**	**Proportion**
I	127	73.8%
II	20	11.6%
III	17	9.9%
IV	8	4.7%
**Total**	**172**	

**Table 3B: England data, 2015-2017, from NHS England [3]**		
**Stage**	**Number of patients**	**Proportion**
I	26530	69.0%
II	8392	21.8%
III	2565	6.7%
IV	977	2.5%
**Total**	**38464**	

**Table 3C: Wales data, 2016-2017, from Public Health Wales [4]**		
**Stage**	**Number of patients**	**Proportion**
I	1031	66.4%
II	373	24.0%
III	105	6.8%
IV	43	2.8%
**Total**	**1552**	

© 2024 Maul LV et al. *JAMA Network Open.*

eTable 4: Data on melanoma incidence before and during the pandemic for Switzerland and Hungary

**Table 4A: Switzerland data – University Hospital Basel, AJCC 7th edition staging**			
**Stage**	**2017-2019**	**2020**	**2021**
I	142	39	54
II	52	16	24
III	28	23	11
IV	4	5	7
**Total**	**226**	**83**	**96**

**Table 4B: Switzerland data – University Hospital Basel, T categories**			
**Stage**	**2017-2019**	**2020**	**2021**
I	99	29	39
II	54	18	24
III	40	13	14
IV	29	16	13
**Total**	**222**	**76**	**90**

**Table 4C: Switzerland data – University Hospital Basel, AJCC 8th edition staging**			
**Stage**	**2017-2019**	**2020**	**2021**
I	142	38	55
II	52	16	23
III	28	24	11
IV	4	5	7
**Total**	**226**	**83**	**96**

© 2024 Maul LV et al. *JAMA Network Open.*

Table 4D: Hungary data – University Hospital of Szeged, AJCC 7th edition staging			
**Stage**	**2019**	**2020**	**2021**
I	127	71	127
II	20	26	30
III	17	21	21
IV	8	5	4
**Total**	**172**	**123**	**182**

Table 4E: Hungary data – University Hospital of Szeged, T categories			
**Stage**	**2019**	**2020**	**2021**
I	125	65	124
II	28	22	24
III	14	13	19
IV	18	28	20
**Total**	**185**	**128**	**187**

Table 4F: Hungary data – University Hospital of Szeged, AJCC 8th edition staging			
**Stage**	**2019**	**2020**	**2021**
I	127	70	126
II	20	26	30
III	19	22	23
IV	6	5	3
**Total**	**172**	**123**	**182**

© 2024 Maul LV et al. *JAMA Network Open.*

Supplementary Table 5: Various characteristics and indicators for each country, along with estimated direct and indirect costs, and years of life lost

Country	ISO2 code	ISO3 code	Membership	Population (millions, 2020-21)	PPP	GDP per capita (international $, 2019)	Health Expenditure (HE) per capita (international $,	GNI per capita (international $, 2019)	Melanoma incidence rate (per 100,000)	Estimated number of melanoma incidences
Austria	AT	AUT	EU	8.946	85.46	58,649.67	5,879.10	58,940.00	23.2	2,075.47
Belgium	BE	BEL	EU	11.556	84.84	54,709.47	5,404.92	55,370.00	28.5	3,293.46
Bulgaria	BG	BGR	EU	6.870	39.99	24,579.22	1,633.80	24,180.00	9.0	618.30
Croatia	HR	HRV	EU	4.014	49.41	30,245.98	1,876.10	29,790.00	16.8	674.35
Cyprus	CY	CYP	EU	0.896	68.14	41,514.51	2,624.85	39,850.00	8.7	77.95
Czech Republic	CZ	CZE	EU	10.730	54.62	43,005.55	3,040.52	40,360.00	24.3	2,607.39
Denmark	DK	DNK	EU	5.840	99.80	60,334.81	5,794.26	62,120.00	50.0	2,920.00
Estonia	EE	EST	EU	1.329	61.10	38,819.34	2,427.63	37,940.00	21.6	287.06
Finland	FI	FIN	EU	5.531	94.48	51,556.53	4,457.17	51,800.00	37.2	2,057.53
France	FR	FRA	EU	65.302	81.78	49,377.13	5,250.45	50,400.00	24.3	15,868.39
Germany	DE	DEU	EU	83.287	83.14	55,891.20	6,098.20	57,410.00	39.0	32,481.93
Greece	GR	GRC	EU	10.668	62.04	30,869.15	2,340.17	30,620.00	12.3	1,312.16
Hungary	HU	HUN	EU	9.767	49.28	33,956.82	2,115.19	33,070.00	15.1	1,474.82
Iceland	IS	ISL	EFTA	0.370	114.65	60,081.85	5,113.22	59,590.00	14.7	54.39
Ireland	IE	IRL	EU	5.041	90.32	89,431.40	5,896.69	69,190.00	28.5	1,436.69
Italy	IT	ITA	EU	60.185	74.84	44,850.93	3,624.08	45,240.00	25.1	15,132.55
Latvia	LV	LVA	EU	1.903	55.60	32,002.76	1,895.78	31,590.00	12.4	235.97
Lithuania	LT	LTU	EU	2.796	50.45	38,765.00	2,312.96	37,420.00	19.1	534.04
Luxembourg	LU	LUX	EU	0.638	94.81	120,962.19	6,047.82	74,310.00	22.8	145.46
Malta	MT	MLT	EU	0.522	64.55	46,766.77	3,897.33	43,240.00	11.9	62.12
Netherlands	NL	NLD	EU	17.458	87.94	59,469.08	5,634.53	59,700.00	48.5	8,467.13
Norway	NO	NOR	EFTA	5.421	110.95	68,345.07	6,818.35	70,330.00	32.8	1,778.09
Poland	PL	POL	EU	37.928	45.96	34,151.79	2,015.29	32,790.00	9.7	3,679.02
Portugal	PT	PRT	EU	10.269	63.15	36,871.84	3,242.35	35,940.00	10.6	1,088.51
Romania	RO	ROU	EU	19.317	39.91	32,299.97	1,576.30	31,840.00	7.9	1,526.04
Slovakia	SK	SVK	EU	5.465	59.20	32,557.19	2,179.54	31,980.00	15.2	830.68
Slovenia	SI	SVN	EU	2.104	62.97	41,193.84	3,158.39	40,530.00	35.2	740.61
Spain	ES	ESP	EU	47.154	70.08	42,185.59	3,576.49	42,250.00	12.5	5,894.25
Sweden	SE	SWE	EU	10.613	93.86	55,068.77	5,828.41	56,670.00	42.1	4,468.07
Switzerland	CH	CHE	EFTA	8.709	116.67	73,114.47	8,113.94	73,620.00	35.6	3,097.34
United Kingdom	UK	GBR	CTA	67.423	87.30	48,513.50	4,619.57	47,620.00	21.9	14,783.83
**Europe (aggregate)**				**528.052**					**24.6**	**129,703.60**

© 2024 Maul LV et al. *JAMA Network Open.*

Country	Years of life lost			Net additional direct costs ($), 17% upstaging rate (millions)			Net additional indirect costs ($), 17% upstaging rate (millions)		
	Years of life with disability (YLD)	Years of life lost (YLL)	Disability-adjusted life years (DALY)	Estimate	Lower bound of 95% CI	Upper bound of 95% CI	Estimate	Lower bound of 95% CI	Upper bound of 95% CI
Austria	245.79	1,783.60	2,029.40	7.68	4.11	13.19	132.77	119.72	146.42
Belgium	390.03	2,830.31	3,220.34	11.52	6.17	19.75	197.92	178.47	218.28
Bulgaria	73.22	531.35	604.57	0.90	0.49	1.53	16.23	14.63	17.90
Croatia	79.86	579.52	659.38	1.19	0.65	2.02	21.80	19.66	24.05
Cyprus	9.23	66.99	76.22	0.19	0.10	0.32	3.37	3.04	3.72
Czech Republic	308.78	2,240.72	2,549.50	6.21	3.35	10.60	114.22	102.99	125.96
Denmark	345.81	2,509.37	2,855.17	11.41	6.13	19.55	196.87	177.52	217.12
Estonia	34.00	246.70	280.69	0.64	0.35	1.09	11.82	10.66	13.04
Finland	243.67	1,768.19	2,011.85	6.85	3.70	11.71	115.68	104.31	127.57
France	1,879.24	13,636.86	15,516.10	51.98	27.82	89.21	868.03	782.71	957.29
Germany	3,846.72	27,914.09	31,760.81	118.42	63.22	203.48	2023.96	1825.03	2232.08
Greece	155.39	1,127.64	1,283.03	2.61	1.42	4.44	43.61	39.32	48.09
Hungary	174.66	1,267.42	1,442.08	2.82	1.53	4.81	52.94	47.73	58.38
Iceland	6.44	46.74	53.18	0.21	0.11	0.36	3.52	3.17	3.88
Ireland	170.14	1,234.65	1,404.79	6.34	3.42	10.84	107.89	97.28	118.98
Italy	1,792.09	13,004.50	14,796.60	42.14	22.78	71.99	743.03	670.00	819.44
Latvia	27.95	202.79	230.73	0.44	0.24	0.76	8.09	7.30	8.92
Lithuania	63.24	458.94	522.18	1.12	0.61	1.91	21.69	19.56	23.92
Luxembourg	17.23	125.01	142.23	0.74	0.40	1.26	11.73	10.58	12.94
Malta	7.36	53.38	60.74	0.17	0.09	0.29	2.92	2.63	3.22
Netherlands	1,002.73	7,276.42	8,279.15	31.43	16.86	53.87	548.63	494.71	605.05
Norway	210.57	1,528.04	1,738.61	7.91	4.24	13.56	135.73	122.39	149.68
Poland	435.69	3,161.65	3,597.34	6.85	3.73	11.67	130.93	118.06	144.40
Portugal	128.91	935.44	1,064.35	2.53	1.37	4.34	42.46	38.29	46.83
Romania	180.72	1,311.44	1,492.16	2.57	1.41	4.37	52.74	47.55	58.16
Slovakia	98.37	713.86	812.24	1.65	0.90	2.80	28.83	26.00	31.80
Slovenia	87.71	636.46	724.17	1.82	0.98	3.11	32.58	29.38	35.93
Spain	698.04	5,065.36	5,763.39	15.55	8.39	26.59	270.29	243.72	298.08
Sweden	529.14	3,839.74	4,368.88	16.46	8.81	28.23	274.82	247.81	303.08
Switzerland	366.81	2,661.77	3,028.58	15.01	8.02	25.79	247.49	223.16	272.94
United Kingdom	1,750.80	12,704.82	14,455.62	46.85	25.20	80.21	764.10	689.00	842.67
**Europe (aggregate)**	**15,360.34**	**111,463.75**	**126,824.09**	**422.21**	**226.56**	**723.67**	**7226.69**	**6516.38**	**7969.78**

© 2024 Maul LV et al. *JAMA Network Open.*

Country	Total additional costs ($), 17% upstaging rate (millions)			Additional costs as fraction of HE, 17% upstaging rate		
	Estimate	Lower bound of 95% CI	Upper bound of 95% CI	Direct	Indirect	Total
Austria	140.45	126.60	154.98	0.015	0.252	0.267
Belgium	209.44	188.79	231.14	0.018	0.317	0.335
Bulgaria	17.12	15.45	18.89	0.008	0.145	0.153
Croatia	22.99	20.74	25.36	0.016	0.290	0.305
Cyprus	3.56	3.21	3.93	0.008	0.143	0.151
Czech Republic	120.42	108.63	132.84	0.019	0.350	0.369
Denmark	208.29	187.76	229.82	0.034	0.582	0.616
Estonia	12.46	11.25	13.74	0.020	0.366	0.386
Finland	122.53	110.43	135.22	0.028	0.469	0.497
France	920.02	828.88	1016.19	0.015	0.253	0.268
Germany	2142.38	1930.68	2364.59	0.023	0.398	0.422
Greece	46.22	41.65	51.01	0.010	0.175	0.185
Hungary	55.76	50.31	61.49	0.014	0.256	0.270
Iceland	3.73	3.36	4.12	0.011	0.186	0.197
Ireland	114.23	102.96	126.07	0.021	0.363	0.384
Italy	785.17	708.11	866.22	0.019	0.341	0.360
Latvia	8.54	7.70	9.41	0.012	0.224	0.237
Lithuania	22.81	20.59	25.16	0.017	0.335	0.353
Luxembourg	12.47	11.23	13.78	0.019	0.304	0.323
Malta	3.08	2.78	3.40	0.008	0.143	0.152
Netherlands	580.06	522.88	640.00	0.032	0.558	0.590
Norway	143.64	129.47	158.52	0.021	0.367	0.389
Poland	137.78	124.32	151.94	0.009	0.171	0.180
Portugal	45.00	40.55	49.67	0.008	0.128	0.135
Romania	55.31	49.93	60.95	0.008	0.173	0.182
Slovakia	30.48	27.49	33.62	0.014	0.242	0.256
Slovenia	34.40	31.03	37.95	0.027	0.490	0.518
Spain	285.84	257.68	315.37	0.009	0.160	0.169
Sweden	291.27	262.42	321.70	0.027	0.444	0.471
Switzerland	262.50	236.49	290.03	0.021	0.350	0.371
United Kingdom	810.95	730.65	895.93	0.015	0.245	0.260
**Europe (aggregate)**	**7648.89**	**6894.03**	**8441.39**	**0.018**	**0.314**	**0.333**

© 2024 Maul LV et al. *JAMA Network Open.*

eTable 6: Estimated upstaging rates for Switzerland and Hungary

Table 6A: Rates (%) based on AJCC 7th edition staging		
	**2020**	**2021**
Switzerland	18.0	15.8
Hungary	16.6	0.4

Table 6B: Rates (%) based on AJCC 8th edition staging		
	**2020**	**2021**
Switzerland	18.3	15.5
Hungary	16.9	0.5

Table 6C: Rates (%) based on T categories		
	**2020**	**2021**
Switzerland	17.5	14.7
Hungary	17.0	0.1

© 2024 Maul LV et al. *JAMA Network Open.*

eMethods

1. Input Data

Direct treatment costs were defined as total expenditures of national health system services including out- and inpatient care, diagnostics and costs due to medical treatment in the adjuvant and palliative setting. Indirect treatment costs were calculated using Disability-Adjusted Life Years (DALYs) to assess the total burden of melanoma. One DALY equals one year of healthy life lost, and can be calculated as the sum of the Years of Life Lost (YLL) due to premature mortality in the population and the Years Lost due to Disability (YLD) for people living with a health condition or its consequences [5].

For indirect cost calculation, we used previously published data based on DALY per person per melanoma stage from a cohort of 5,928 melanoma patients recorded by the Belgian Cancer Registry for the incidence years 2009–2011 [2] (eTable 2). We used gross domestic product (GDP), gross national income (GNI), health expenditure (HE) and purchasing power parity (PPP) as country-level economic indicators, obtained by the World Bank (eTable 5). Values for 2019 were taken as baseline, since many economies experienced sudden affection by the pandemic during 2020-2021, influencing the values of these economic parameters. PPP is expressed as a number with reference to the international US dollar ($) set at 100. For comparability and the ability to combine across countries, we followed Krensel et al. [6] in using a version of the indicators scaled to the international US dollar (using PPP for each country and other factors such as exchange rates). All calculated monetary costs were finally reported in US dollar ($), thus any Euro (€) numbers were converted to USD using a conversion rate of 1 Euro to 1.11 USD, the average exchange value during the year 2020.

We used melanoma incidence data for the 28 member countries of the EU (including the United Kingdom, which left the EU due to Brexit on 1st January 2021), and 3 countries that are part of the EFTA (Switzerland, Norway, and Iceland), for the period 2010-20, obtained by the European Cancer Information System (ECIS) [7]. These data originated from cancer registries of the individual countries. Generally, the most recent available value was taken. However, when the reported incidence of 2020 was lower than the previous year (possible due to under-reporting in the lockdown), the previous year's value was used.

Population per country numbers were taken from the International Monetary Fund [8]. The upstaging rate of melanoma patients during COVID-19 lockdown was estimated with real-life pre- and peri-pandemic stagewise incidence data for two countries (Switzerland and Hungary). We thus performed calculations for European countries primarily with the estimated 17% increase (see main text), as well as a range of scenarios from 8% to 45% increase based on recent publications [9,10]. We did not include attrition in our model, as the expected proportion of people to drop out of the calculations due to death during the few months of the lockdown period is fairly low. [1,11,12]

© 2024 Maul LV et al. *JAMA Network Open.*

2. Model Development

2.1. Estimation of additional indirect costs (as life-years lost) due to upstaging

eTable 7 below and Figures 2B and 2C explain the calculation of additional cost incurred due to upstaging (ACU). Cost values corresponding to stage are indicated with a suffix. In addition to stage (k), the number of patients per stage (n_k) and cost per person per stage (c_k) were used from a cohort. The cost can be either direct, (Euro, €), indirect (DALY), or years of life lost from mortality (YLL)).

The proportion of patients at each stage (p_k), was calculated by dividing n_k by the total number of patients (n) in the cohort. If the upstaging rate parameter is u, it means that a proportion u of patients in any stage moved to the next melanoma stage during this period. Therefore, $p_1 \times u$ patients would move from stage I to II during lockdown. The corresponding cost increase would be $c_2 - c_1$, so the ACU incurred specifically for stage I melanoma patients would be $p_1 \times u \times (c_2 - c_1)$. These values can be added for stages I to III to obtain the aggregate ACU:

$$a = u \times [p_1 \times (c_2 - c_1) + p_2 \times (c_3 - c_2) + p_3 \times (c_4 - c_3)]$$

Equation 1

Patients in stage IV cannot upstage any further.

eTable 7. Schematic overview of the calculation process for additional cost incurred due to upstaging.

Stage	Number of patients	Proportion	Proportion upstaged	Cost per person per stage	Cost increases due to upstaging	Additional cost
I	n_1	$p_1 = n_1 / n$	$p_1 \times u$	c_1	$c_2 – c_1$	$p_1 \times u \times (c_2 – c_1)$
II	n_2	$p_2 = n_2 / n$	$p_2 \times u$	c_2	$c_3 – c_2$	$p_2 \times u \times (c_3 – c_2)$
III	n_3	$p_3 = n_3 / n$	$p_3 \times u$	c_3	$c_4 – c_3$	$p_3 \times u \times (c_4 – c_3)$
IV	n_4	$p_4 = n_4 / n$	-	c_4	-	-
Total	n	1	-	-	-	a

The ACU is a weighted average of the increase in stage-wise treatment costs, weighted by stage-wise incidence rates.

© 2024 Maul LV et al. *JAMA Network Open.*

The DALY cost values (in years) were used as the cost column in the schematic Table to obtain the additional indirect cost due to upstaging (AICU).

This model assumes that the upstaging rate parameter is the same across all of the melanoma stages. This is because more fine-scale estimates of upstaging rates separately for each melanoma stage were not available in the literature[10,11].

2.2. Estimation of additional direct costs due to upstaging

Direct costs (in Euro) from each source were converted to direct costs (in Euro) for each target country by using various economic indicators (GDP, HE, and PPP), resulting in a `standardized` cost value for each country, based on previous models[6].

The converted cost values for each target country were used to calculate additional direct cost due to upstaging (ADCU) in each country, following the same procedure as indirect costs, as shown in Equation 1 and eTable 7.

2.3. Estimating indirect costs' variance

As the cost estimates derived in this process are aggregate estimates of cost increase for entire countries, there are several components of variability that need to be considered, reflecting the inherent uncertainty of such an estimation procedure. We used the probabilistic sensitivity analysis (PSA) method, [2] by modelling the various components of the input data generation process using various statistical distributions, drawing simulated values of input data, and producing simulated estimates of cost increase using these simulated data. We repeated the random simulations 10,000 times and obtained a 95% confidence interval (CI) by taking the 2.5% quantile of the simulated cost values as the lower bound and the 97.5% quantile as the upper bound of the interval.

First component of variability: The total number of patients with each melanoma stage in a country (N_k) are random variables, whose proportions could be assumed to follow the incidence rate estimates of each stage (p_k, obtained from registries). We modelled the set of numbers (n_k) with a multinomial model, which means that any specific count n_k will follow a binomial model with parameters (n, p_k), with mean $N \times p_k$ and variance $N \times p_k \times (1 - p_k)$. New data was generated by simulating random multinomial data using these parameters. This component represents the statistical variability of the actual number of melanoma patients at each stage.

Second: We noted that the YLD and YLL estimates for each melanoma stage provided in [2] were average values across the patients in each stage. The variances corresponding to these estimates were not provided, but sensitivity analyses were performed for YLD and YLL per melanoma, and a 95% confidence interval was provided. Its width was used to calculate the standard error (SE) of the variable. [2] Log-normal distributions were used to model the distribution of YLD and YLL values, the parameters of the distributions estimated

from the average and SE obtained above. New random values of YLD and YLL were simulated from these distributions and used in cost calculations. This component represents the statistical variability inherent when the YLD and YLL estimates in the publication [2] were obtained.

These simulated incidence values from various sources, and the simulated YLD and YLL values, were used in the cost estimation model in the same way to obtain a simulated estimate of additional indirect cost. For example, in any one particular simulation, the estimates obtained by using simulated incidence proportions from each source were averaged. 10,000 such simulated indirect costs were used to obtain the 95% CI.

2.4. Estimating direct costs' variance

Estimation of a variance of direct cost increase due to upstaging was similar to the process for indirect costs, using the PSA procedure. The first component of variability, representing statistical variability of the different sources of stage distribution at diagnosis (incidence) rates, was procedurally identical.

The second component of variability was also equivalent between direct and indirect costs. For the direct cost values for Italy [1], average and SE of direct treatment costs per stage were available, so a log-normal model was used to generate new random cost values for each stage. In the case of Switzerland, treatment cost values for individual patients at each stage were available, so random resampling (with replacement) within each stage (i.e. stratified resampling) was used to generate a new set of cost values, and their average was used.

As the third component we looked at the variability of direct cost estimates per melanoma stage. While we used the average direct cost values from each source in calculating cost increases, in addition, the standard deviation (SD) of the direct cost value for each melanoma stage was available from the Italian source. These variances were fed into the formula used to calculate additional cost due to upstaging to obtain a variance of that expression representing this variability in direct costs.

These simulated incidence values from various sources, and the simulated direct cost values, were used in the cost estimation model in the same way to obtain a simulated estimate of additional direct cost. The simulations were performed 10,000 times to obtain the CI.

2.5. Estimating upstaging rate from incidence numbers

Stage-wise incidence numbers from two centers, Switzerland and Hungary, were available before and during the pandemic, which allowed us to estimate upstaging rates from these incidences. For Switzerland, data from 2017-2019 was taken as pre-pandemic, whereas for Hungary only 2019 was available. For during-pandemic numbers, data for 2020 and 2021 were used separately. In each case, tumor (T) stage-specific incidence numbers were taken following two approaches: AJCC (7th edition) [12] and primary cutaneous melanoma T category stratification.

© 2024 Maul LV et al. *JAMA Network Open.*

eTable 8. Schematic overview of the calculation process for upstaging rate.

Stage	Number of patients pre-pandemic	Proportion	Proportion upstaged	Proportion during pandemic after upstaging	Number of patients during pandemic	Cost per person per stage
I	n_1	$p_1 = n_1 / n$	$p_1 \times u$	$q_1 = p_1 \times (1 - u)$	m_1	c_1
II	n_2	$p_2 = n_2 / n$	$p_2 \times u$	$q_2 = p_2 \times (1 - u) + p_1 \times u$	m_2	c_2
III	n_3	$p_3 = n_3 / n$	$p_3 \times u$	$q_3 = p_3 \times (1 - u) + p_2 \times u$	m_3	c_3
IV	n_4	$p_4 = n_4 / n$	-	$q_4 = p_4 + p_3 \times u$	m_4	c_4
Total	n	1	-	1	m	-

The number of patients per stage (n_k) before the pandemic in a location was compared to the number of patients in a year during the pandemic (m_k) to estimate the upstaging rate parameter (u). This was done using the statistical method of maximum likelihood estimation. The likelihood is the probability of observing the number of patients during the pandemic (m_k) under the probability model assumed to underly the scenario. As before, the multinomial model was assumed, so the numbers m_k follow a multinomial distribution with total m and proportions q_k. With this model, the likelihood is proportional to:

$$L = \prod_{k=1}^{4} {q_k}^{m_k}$$

Equation 2

So the log-likelihood is proportional to

$$l = m_1 \log[n_1(1-u)] + m_2 \log[n_2(1-u) + n_1 u] + m_3 \log[n_3(1-u) + n_2 u] + m_4 \log[n_4 + n_1 u]$$

Equation 3

For given values of n_k and m_k, this expression is numerically maximized to find the best estimate of the upstaging rate parameter u.

2.6. Model validation from incidence numbers

The model used in this study to calculate the increase in costs due to upstaging is based on the upstaging rate parameter. This is a relatively simple and parsimonious model, since the user can easily obtain an idea of the range of additional costs by only varying a single parameter over a range of estimates, and is therefore used in the literature to model the effect of COVID-19 lockdowns [6]. However, this involves making certain assumptions, such as the rate being the same across all stages [6]. These assumptions are useful given the sparsity of data, especially for the higher stages, which makes estimation of separate rates for each stage less reliable.

© 2024 Maul LV et al. *JAMA Network Open.*

We estimate an upstaging rate of 17% from real-life incidence data during the pandemic from two locations, which is used for the majority of additional cost estimates reported in the study. This estimation is also based on a model, described in the previous section.

We validate the use of these models by calculating cost increase directly by using real-life incidence numbers and cost values (eTable 6), without any modelling assumptions. Before the pandemic, the average cost for treatment of a patient, aggregated across melanoma stages, would've been

$$C_{before} = \sum_{k=1}^{4} c_k p_k = \frac{1}{n}\sum_{k=1}^{4} c_k n_k$$

Equation 4

During the pandemic, this cost would become

$$C_{during} = \frac{1}{m}\sum_{k=1}^{4} c_k m_k$$

Equation 5

Therefore, the additional cost increase due to the pandemic is

$$C_{additional} = C_{during} - C_{before} = \sum_{k=1}^{4} c_k \left(\frac{n_k}{n} - \frac{m_k}{m}\right)$$

Equation 6

This calculation (Equation 6) is based entirely on observed real-life numbers without any modelling assumptions. The value calculated this way is compared in the main text with the cost estimate based on the upstaging rate-based cost model (Equation 1). In that modelling calculation, the upstaging rate parameter used was calculated using the same real-life incidence numbers, as described above. We show in the text that the two estimates are very close, and one falls within the confidence interval of the other, thereby validating the use of the models.

© 2024 Maul LV et al. *JAMA Network Open.*

eReferences

1. Buja A, Sartor G, Scioni M, et al. Estimation of Direct Melanoma-related Costs by Disease Stage and by Phase of Diagnosis and Treatment According to Clinical Guidelines. *Acta Derm Venereol*. Feb 7 2018;98(2):218-224. doi:10.2340/00015555-2830
2. Tromme I, Legrand C, Devleesschauwer B, et al. Melanoma burden by melanoma stage: Assessment through a disease transition model. *Eur J Cancer*. Jan 2016;53:33-41. doi:10.1016/j.ejca.2015.09.016
3. National Disease Registration Service (NDRS). Staging data in England. Accessed July 15, 2023, https://www.cancerdata.nhs.uk/stage_at_diagnosis
4. NHS Wales. Cancer Incidence in Wales, 2002-2019. Accessed July 15, 2023, https://phw.nhs.wales/services-and-teams/welsh-cancer-intelligence-and-surveillance-unit-wcisu/cancer-reporting-tool-official-statistics/cancer-incidence/cancer-incidence-documents/data-tables/
5. WHO. Global Health Estimates: Life expectancy and leading causes of death and disability. Accessed July 15, 2023, https://www.who.int/data/gho/data/themes/mortality-and-global-health-estimates
6. Krensel M, Schafer I, Augustin M. Cost-of-illness of melanoma in Europe - a modelling approach. *J Eur Acad Dermatol Venereol*. Mar 2019;33 Suppl 2:34-45. doi:10.1111/jdv.15308
7. European Commission. ECIS - European Cancer Information System. Accessed July 15, 2023, https://ecis.jrc.ec.europa.eu
8. International Monetary Fund. World Economic Outlook Database: April 2021. Accessed July 15, 2023, https://www.imf.org/en/Publications/WEO/weo-database/2021/April/weo-report?c=512,914,612,614,311,213,911,314,193,122,912,313,419,513,316,913,124,339,638,514,218,963,616,223,516,918,748,618,624,522,622,156,626,628,228,924,233,632,636,634,238,662,960,423,935,128,611,321,243,248,469,253,642,643,939,734,644,819,172,132,646,648,915,134,652,174,328,258,656,654,336,263,268,532,944,176,534,536,429,433,178,436,136,343,158,439,916,664,826,542,967,443,917,544,941,446,666,668,672,946,137,546,674,676,548,556,678,181,867,682,684,273,868,921,948,943,686,688,518,728,836,558,138,196,278,692,694,962,142,449,564,565,283,853,288,293,566,964,182,359,453,968,922,714,862,135,716,456,722,942,718,724,576,936,961,813,726,199,733,184,524,361,362,364,732,366,144,146,463,528,923,738,578,537,742,866,369,744,186,925,869,746,926,466,112,111,298,927,846,299,582,487,474,754,698,&s=LP,&sy=2021&ey=2021&ssm=1&scsm=1&scc=1&ssd=1&ssc=0&sic=1&sort=country&ds=.&br=1
9. Degeling K, Baxter NN, Emery J, et al. An inverse stage-shift model to estimate the excess mortality and health economic impact of delayed access to cancer services due to the COVID-19 pandemic. *Asia Pac J Clin Oncol*. Feb 10 2021;doi:10.1111/ajco.13505
10. Tejera-Vaquerizo A, Nagore E. Estimated effect of COVID-19 lockdown on melanoma thickness and prognosis: a rate of growth model. *J Eur Acad Dermatol Venereol*. Aug 2020;34(8):e351-e353. doi:10.1111/jdv.16555
11. Degeling K, Baxter NN, Emery J, et al. An inverse stage-shift model to estimate the excess mortality and health economic impact of delayed access to cancer services due to the COVID-19 pandemic. *Asia Pac J Clin Oncol*. Aug 2021;17(4):359-367. doi:10.1111/ajco.13505
12. Balch CM, Gershenwald JE, Soong SJ, et al. Final version of 2009 AJCC melanoma staging and classification. *J Clin Oncol*. Dec 20 2009;27(36):6199-206. doi:10.1200/jco.2009.23.4799

© 2024 Maul LV et al. *JAMA Network Open.*
