# Supplementary material for: Health Economic Consequences Associated With COVID-19–Related Delay in Melanoma Diagnosis in Europe
Source: JAMA Netw Open. 2024 Feb 16;7(2):e2356479. doi: 10.1001/jamanetworkopen.2023.56479 (PMC10873772; doi:10.1001/jamanetworkopen.2023.56479)
Supplement: Supplement 2. — Data Sharing Statement [file jamanetwopen-e2356479-s002.pdf]

Data Sharing Statement

Maul. Health Economic Consequences Associated With COVID-19–Related Delay in Melanoma Diagnosis in Europe. *JAMA Netw Open*. Published February 16, 2024. doi:10.1001/jamanetworkopen.2023.56479

Data
Data available: Yes
Data types: Deidentified participant data
How to access data: Elisabeth.roider@usb.ch
When available: With publication

Supporting Documents
Document types: None

Additional Information
Who can access the data: anyone requesting the data
Types of analyses: for any purpose
Mechanisms of data availability: with a signed data access agreement
